# Influence of Diet, Sex, and Viral Infections on the Gut Microbiota Composition of *Spodoptera exigua* Caterpillars

**DOI:** 10.3389/fmicb.2020.00753

**Published:** 2020-05-06

**Authors:** María Martínez-Solís, María Carmen Collado, Salvador Herrero

**Affiliations:** ^1^Estructura de Recerca Interdisciplinar en Biotecnologia i Biomedicina (ERI BIOTECMED), Departamento de Genética, Universitat de València, Valencia, Spain; ^2^Instituto de Agroquímica y Tecnología de Alimentos, Consejo Superior de Investigaciones Científicas (IATA-CSIC), Valencia, Spain

**Keywords:** lepidoptera, microbiota, *Spodoptera exigua*, 16S rRNA, viral infection

## Abstract

The gut microbiota plays essential roles in processes related with metabolism, physiology, and immunity in all organisms, including insects. In the present work, we performed a broad analysis of the *Spodoptera exigua* gut microbiota, a major agricultural pest. We analyzed the influence of multiple parameters such as diet, geographic location, sex, or viral infections on *S. exigua* caterpillar gut microbiota composition. Our study revealed a high variability in bacterial composition among individuals, and a major influence of environmental bacteria (including those acquired through diet) on the gut microbiota composition, supporting previous studies that claim resident microbiota are lacking in caterpillars. Previous studies with laboratory-reared insects showed that changes in caterpillar gut bacterial composition affect the insecticidal properties of entomopathogenic viruses and bacteria. Our study revealed different microbiota composition in field insects carrying a natural viral infection with *Spodoptera exigua nucleopolyhedrovirus* (SeMNPV) and/or *Spodoptera exigua iflavirus 1* (SeIV1). Few taxa can be specifically associated with the infection, suggesting microbiota influence the infective process of these natural pathogens, and providing new strategies for insect pest management.

## Introduction

The Lepidoptera *Spodoptera exigua* (Hübner) (Noctuidae) is an important polyphagous agricultural pest, polyphagous and worldwide distributed. Traditionally, chemical insecticides have been used to control this pest but, extensive use over a long time period has led to the emergence of resistant populations (Brewer and Trumble, 1989; Moulton et al., 2000; Osorio et al., 2008; Ahmad and Arif, 2010; Che et al., 2013). Therefore, biological insecticides such as baculovirus and *Bacillus thuringiensis* (Bt) represent an effective and safer alternative for pest control.

Gut microbiota is described as the complex community of microorganisms living in the digestive tracts of human and other animals in a symbiotic relationship. Many animals, including insects, are colonized by microbial symbionts, which can regulate host processes related with development, immunity, and metabolism (Dillon and Dillon, 2004; Engel and Moran, 2013). Most caterpillars are herbivorous, and their gut bacteria can play important roles in nutrition and host adaptation. For instance, gut microbiota in caterpillars can contribute to plant cell wall digestion (Mason et al., 2015; Xia et al., 2017), detoxification of toxic compounds synthesized by the plants they feed on (Shao et al., 2011; Hammer and Bowers, 2015), or even suppress plant defense mechanisms (Acevedo et al., 2017). In recent years, researchers investigated if caterpillars depend on their gut microbes for feeding and/or development, but this question remains unclear. While some studies have shown that diet affects the insect microbiome composition (Berman et al., 2018; Jones et al., 2019), others have reported that diet does not have a clear effect on gut microbiota composition (Whitaker et al., 2016; Hammer et al., 2017). Moreover, the great variability usually observed in lepidopteran bacterial composition also indicates that many factors influence the final gut bacterial composition of these insects (reviewed in Mereghetti et al., 2017).

Furthermore, the Lepidoptera gut microbiota seems to influence the host interaction with pathogenic microorganisms. Several studies on different lepidopteran species revealed changes in gut microbiota composition after intoxication with Bt toxins (Broderick et al., 2004; Caccia et al., 2016), and insect susceptibility alteration to Bt and its toxins (Broderick et al., 2004) due to gut microbiota changes. In addition, Xia et al. (2013) suggested that certain bacterial taxa can confer Bt-resistance in *Plutella xylostella* larvae. In the case of *S. exigua*, our previous studies showed an increase in Bt tolerance associated with an increase in the gut bacterial load (Hernández-Martínez et al., 2010). Regarding viral pathogens such as baculovirus, a previous study from our laboratory revealed an increase in gut bacterial load after infection of a laboratory-reared *S. exigua* colony with its baculovirus, *Spodoptera exigua multiple nucleopolyhedrovirus* (SeMNPV) (Jakubowska et al., 2013). Such change in bacterial load was associated with an increase in baculovirus virulence, pathogenicity, and dispersion.

Baculoviruses are DNA viruses highly specific against different invertebrate species which cause larval death after viral ingestion. In addition to the oral infection caused by baculovirus, the virus can asymptomatically persist on the insects and be vertically transmitted to the offspring (Virto et al., 2014). In the case of *S. exigua*, 54% of field adult insects are covertly infected with its baculovirus, SeMNPV (Virto et al., 2014). In addition, some field insects can also carry a second covert infection with RNA viruses from the *Iflaviridae* family, which apparently do not cause lethal infection. *Spodoptera exigua Iflavirus 1* (SeIV1) (Millán-Leiva et al., 2012) was present in about 13.1% of field insects and 6.1% of insects were simultaneously infected with SeMNPV and SeIV1 (Virto et al., 2014). These covertly infectious viruses can be activated by different stress factors, leading to a lethal infection and finally killing the insect.

Based on the previous observations mentioned above, we hypothesized the possible role of gut microbiota composition in modulating *S. exigua* interaction with its naturally occurring viruses in the field. In the present work, as a first step in this investigation, we characterized the gut microbiota composition of laboratory and field collected *S. exigua* caterpillar, and studied the influence of dietary regimen, geographic location, and caterpillar sex on the gut microbiota composition. In a second part of the study, we analyzed viral infection interaction and gut microbiota composition, identifying certain bacterial groups which could shape viral-host interaction.

## Materials and Methods

### Insects

In this study, samples came from *S. exigua* larvae with different backgrounds and dietary regimens. Field larvae were obtained from pepper greenhouses located in the Almería province (Spain). Third to fifth instar larvae were collected during September and October 2015 and sent to our laboratory in Valencia (Spain). The insects were sent in individual plastic bottles containing the pepper leaf from where they were collected, and the larvae were dissected and processed immediately upon arrival. The insects reared on standard artificial diet (AD) (Elvira et al., 2010) came from our laboratory colony, which has been maintained for more than 200 generations. The colony is maintained at 25 ± 3°C with 70 ± 5% relative humidity and a 16/8 h (light/dark) photoperiod. We also used *S. exigua* larvae from our laboratory colony fed a plant-based diet (PBD), which was prepared with lyophilized plant leaves in 2% agar and supplemented with 5% AD. For the PBD studies, fourth instar larvae previously reared on AD were transferred to the PBD and reared for 48 h before dissection. In parallel, a group of larvae were kept only on AD and dissected simultaneously. The plants employed for the PBD studies were pepper (dulce de España variety) and two different tomato varieties [Ailsa (A) and Money Maker (MM)]. Plants were grown in greenhouse facilities and harvested before floriation.

The *S. exigua* larvae were dissected and processed as follows. The whole gut (including the gut content) of each individual larva from the field, and pools of three guts per sample from an AD and PBD were dissected and homogenized in Luria-Bertani (LB) medium supplemented with 10% glycerol and frozen at −80°C for DNA extraction. For each sample, half of the remaining body was directly frozen at −80°C for subsequent DNA extraction, and the other half was frozen in RNAzol^®^ RT reagent (Sigma Aldrich, St Louis, MO, United States) at −80°C for RNA extraction.

### DNA Extraction and Sequencing

A fraction of each homogenized gut was used for total DNA extraction using the MasterPure^TM^ Complete DNA & RNA Purification Kit (Epicenter, Madison, WI, United States) according to the manufacturer’s instructions. Purified DNA was quantified in QUBIT and 10 ng/ul were used for amplification and sequencing of the 16S rRNA gene from 77 samples. The amplicon sequencing protocol targets the 16S gene V3 and V4 regions (459 bp), with the primers designed surrounding conserved regions (Klindworth et al., 2013). Following the Illumina amplicon libraries protocol, DNA amplicon libraries were generated using a limited cycle PCR: initial denaturation at 95°C for 3 min, followed by 25 cycles of annealing (95°C 30 s, 55°C 30 s, 72°C 30 s), and extension at 72°C for 5 min, using a KAPA HiFi HotStart ReadyMix (KK2602). Then Illumina sequencing adaptors and dual-index barcodes (Nextera XT index kit v2, FC-131-2001) were added to the amplicon. Libraries were then normalized and pooled prior to sequencing. The pool containing indexed amplicons was then loaded onto the MiSeq reagent cartridge v3 (MS-102-3003) spiked with 10% PhiX control to improve base calling during sequencing, as recommended by Illumina for amplicon sequencing. Sequencing was conducted using a 2 × 300-pb paired-end run on an Illumina MiSeq sequencing system.

### Sex Determination

Field larvae sex was molecularly determined by *kettin* gene copy number relative quantification. *Kettin* is a sex-linked gene without dosage compensation as previously described for *Bombyx mori* (Suzuki et al., 1999), and was recently used to determine sex in different Lepidopteran larvae species (Belousova et al., 2019). In this study, specific primers for *S. exigua kettin* gene amplification were designed (Supplementary Table 1) using the same region published previously for *B. mori* (Koike et al., 2003). Primers were previously validated on thorax DNA obtained from *S. exigua* male and female adults.

Total DNA was extracted from field larvae carcasses using the MasterPure^TM^ Complete DNA & RNA Purification Kit (Epicenter, Madison, WI, United States), and the isolated DNAs (50 ng) were used for *kettin* quantification by quantitative PCR (qPCR) using the specific primers and *ATP synthase* as a reference. The qPCR was performed with 5x HOT FIREPOL EvaGreen qPCR Mix Plus (ROX) (Solis BioDyne, Tartu, Estonia) following standard protocols, and the DNA amplification was measured in a StepOnePlus Real-Time PCR System (Applied Biosystems, Foster City, CA, United States). The relative quantification was represented as 2^–ΔΔCt^ calculated according to the method described in Livak and Schmittgen (2001) and Belousova et al. (2019), were ΔΔCt = (Ct_*kettin*_ – Ct_ATP synthase_) - Avg(Ct_*kettin*_ – Ct_*ATP synthase*_)_female_.

### RNA Extraction and Virus Quantification

The presence of the SeIV1 RNA virus and the active replication of the SeMNPV DNA virus in field insects were determined by qPCR using specific primers (Supplementary Table 1). Total RNA was isolated from the carcass of each larvae using RNAzol^®^ RT reagent (Sigma Aldrich, St Louis, MO, United States) following the manufacturer’s protocol, and used for cDNA synthesis using the PrimeScript RT Reagent kit (TaKaRa Bio Inc., Otsu Shiga, Japan). The qPCR was performed as described in the previous section. The amplification curve of each sample was examined, and ΔRn ≥1 values were considered as positive viral infections.

### Total Bacterial Load Quantification

The total bacterial load was quantified in the field samples by qPCR using 16S rRNA universal primers (Nadkarni et al., 2002). The qPCR was performed with total DNA (50 ng) isolated from the gut of each larvae in a StepOnePlus real-time PCR system (Applied Biosystems, Foster City, CA, United States). The bacterial concentration was calculated by comparison with a standard curve of known bacterial DNA quantities, and then statistically analyzed for viral presence with a Student’s *t-*test (GraphPad Prism version 7.00) as described in Gasmi et al. (2019).

### Data Analysis

Data-mining and statistical analysis were performed with the open-source software QIIME (v. 1.9) (Caporaso et al., 2010) and the online Calypso pipeline (v. 8.84) (Zakrzewski et al., 2017). Quality assessment of obtained reads was carried out with the prinseq-lite program (Schmieder et al., 2011) with defined parameters (i.e., min_length, 50; trim_qual_right, 20; trim_qual_type, mean; trim_qual_window, 20). Paired reads from Illumina sequencing were joined using fastq-join from the ea-tools suite (Aronesty, 2011). Potential chimeric sequences were removed using USEARCH 6.0 available at RDPipeline (Edgar et al., 2011). Filtered and demultiplexed sequences were then processed with QIIME software using default parameters. First, the sequences were clustered into operational taxonomic units (OTUs) using *de novo* OTU picking (pick_de_novo_otus.py script) based on 97% identity and filtering the unassigned, mitochondria, chloroflexi, and cyanobacteria taxa using the QIIME’s filter_taxa_from_otu_table.py script, to visualize the most abundant phyla in all samples. Next, to analyze the samples more deeply to look for microbiotia differences, the generated OTU table was filtered to include only the four most abundant phyla. Bacterial composition was analyzed through the summarize_taxa_through_plots.py script and it was represented as the relative abundance of the 20 most abundant genera in a bar graphic using Excel software. We also identified the core microbiome (compute_core_microbiome.py) as those OTUs present in at least 50% of all samples.

The OTU table including only the most abundant phyla was filtered in Calypso software to remove the samples with less than 1000 reads from the analysis, and the resulting data was transformed by CSS (cumulative-sum scaling)+log with total sum normalization (TSS). Then, canonical correspondence analysis (CCA) was used to estimate differences in microbiota (at the genus level) according to different factors (diet, sex, or viral infections). Alpha diversity and richness were determined at the genus level using the Shannon index and Chao1 index, respectively. The Wilcoxon rank test was employed to identify differentially abundant bacteria associated with viral presence. Spearman correlation analysis using GraphPad Prism (version 7.00) were used to examine the relationship between the total bacterial loads and the diversity or richness in field samples.

## Results

### *S. exigua* Caterpillar Microbiota Compositions

**FIGURE 1:**
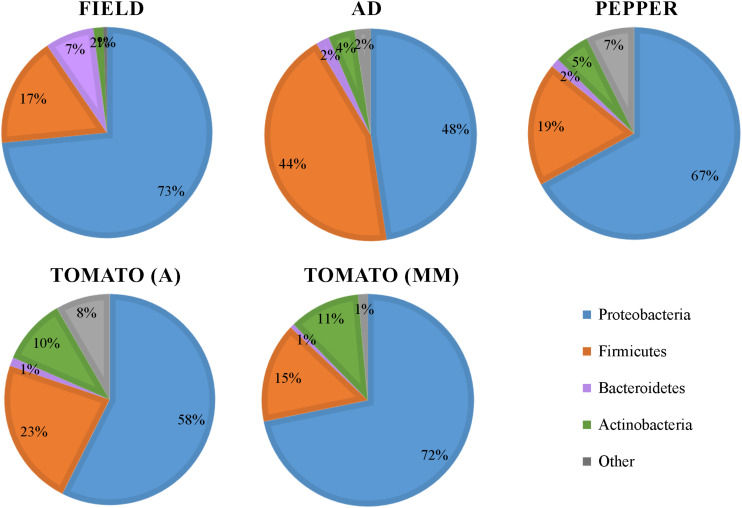
Bacterial phyla distribution in the gut of *S. exigua*. Pie charts represent the relative abundance in percentages of the main phyla found in the gut of *S. exigua* larvae collected on the field from pepper greenhouses and the laboratory colony fed with different diets (AD: artificial diet; A: Ailsa variety; MM: Money Maker variety).

A total of 3,450,084 reads were obtained from 16S rRNA gene Illumina Miseq sequencing of 77 *S. exigua* samples (45 samples from field captures, 15 from AD, and 17 from the PBD) (detailed on Supplementary Tables 2,3). After cleaning and chimera filtration, the remaining 2,795,707 reads led to the identification of 42,601 OTUs. After removing some host *S. exigua* contaminant sequences (designated as unassigned), mitochondria, chloroflexi, and cyanobacteria taxa, 21,197 OTUs remained. These OTUs were assigned mostly to Proteobacteria, Firmicutes, Bacteroidetes, and Actinobacteria phyla (98% total phyla coverage) showing different distribution across the analyzed groups (Figure 1). Proteobacteria was the most abundant phyla in all groups, representing around 50% or more OTUs in the different groups. The phylum Firmicutes was almost as abundant as Proteobacteria in the AD group but decreased to around 20% in the larvae fed PBD group. Bacteroidetes was the least abundant in all groups, although its presence was slightly higher in field samples than from laboratory larvae. Actinobacteria was more abundant in samples from two tomato varieties. Additionally, 8 samples (5 field samples, 1 AD, 1 from tomato (MM), and 1 from pepper) were removed from the analysis, since they contained less than 1000 reads. Although some variability in the richness and Shannon indices between the individual samples was observed (Supplementary Figures 1A,B), rarefaction curves tended to reach a plateau indicating that the sequencing depth was enough to capture the majority of microbial diversity (Supplementary Figure 1C).

### Changes in Gut Microbiota Associated With Different Diets

**FIGURE 2:**
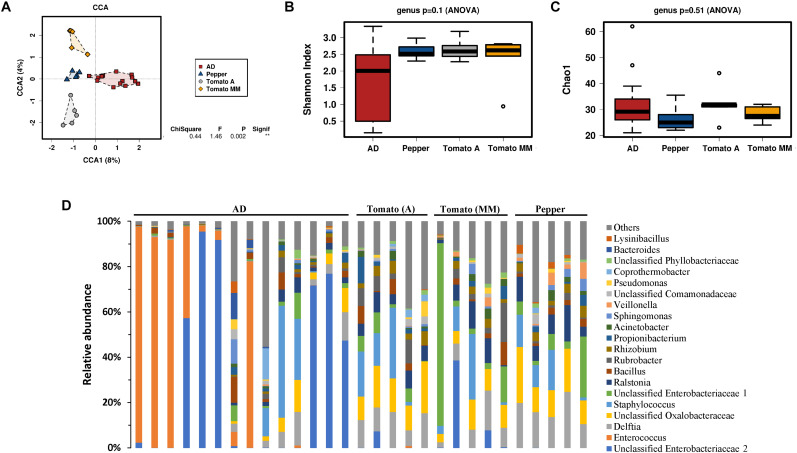
Microbiota of *S. exigua* larvae according the diet. Canonical correspondence analysis (CCA) **(A)**, estimation of the Shannon index diversity **(B)**, the Chao1 index of richness **(C)** and the relative abundance of the 20 most abundant genera in *S. exigua* larvae from the laboratory colony fed with different diets **(D)** at a genus level.

Influence of diet on the bacterial community composition was further characterized according to their genus distribution (Figure 2) by analyzing bacterial composition changes in caterpillar reared on AD, and then transferred to a PBD for 48 h. A multivariate canonical correspondence analysis (CCA) at the genus level revealed significant differences (*P* = 0.002) among the different diet types. Accordingly, changes in diet composition produced a quick shift in gut microbial composition, suggesting that *S. exigua* larvae microbiota composition was strongly influenced by ingested food. Composition differences were observed even among larvae fed (for 48 h) with two different tomato varieties (Figure 2A).

The bacterial diversity measured by the Shannon index at the genus level was almost identical for larvae fed a PBD, showing very little variability among samples (Figure 2B). In contrast, those larvae fed an AD showed an apparent greater variability among each sample. Nevertheless, no significant differences in bacterial diversity were observed between the different diets tested. The Chao1 index for richness estimation also did not show differences between diets (Figure 2C). Further bacterial composition characterization was carried out by relative abundance comparison of the 20 most abundant genera for each sample (Figure 2D). Great genus composition heterogeneity was observed and, consequently, no clear association of specific genera to the different diets could be established.

### Gut Microbiota Composition in Field *S. exigua* Larvae

**FIGURE 3:**
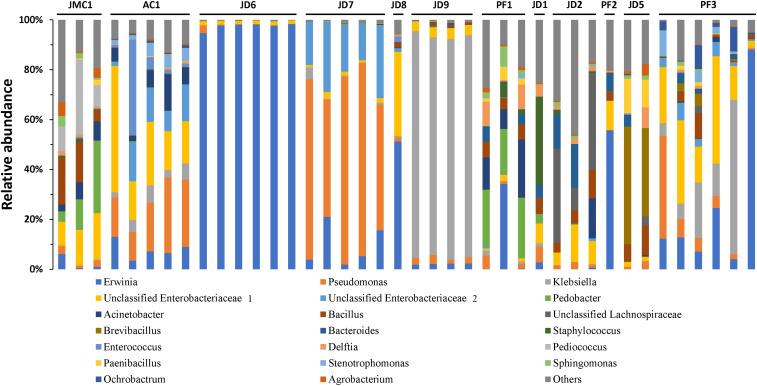
Microbiota composition of *S. exigua* according the location. Relative abundance of the 20 most abundant genera in *S. exigua* larvae from field. The codes of numbers and letters refer to the different greenhouses in which larvae were collected.

*S. exigua* bacterial composition was also evaluated in individual larvae collected from pepper plants from different greenhouses in the Almería province (Spain). Relative abundance analysis of the 20 most abundant genera revealed great similarity among individuals collected from the same greenhouse (Figure 3). However, samples from different greenhouses were highly diverse in composition. These results suggest a major influence of environment (including the ingested plant) on *S. exigua* larvae gut bacterial composition.

### Core Microbiome

A core microbiome was defined as those OTUs present in at least 50% of all samples. Taking all samples individually into account, a core that comprises 20 OTUs belonging to phyla Proteobacteria was identified (Table 1). However, no bacteria (OTU) were found in common for all samples analyzed. In addition, a core microbiome was also analyzed in a more restricted sample group, the field-collected insects. In that case, only one OTU, belonging to the *Pseudomonas* genus, was found in common in all the field individuals. Results obtained with the two sample sets analyzed revealed the lack of a true core microbiome in the *S. exigua* caterpillar, since no OTUs were found in common between all the analyzed samples.

**TABLE 1:** The most common OTUs from the gut microbiota of *S. exigua* larvae.

OTU	Prevalence (%)
p__Bacteroidetes__f__Enterobacteriaceae_1157	92.75
p__Bacteroidetes__f__Enterobacteriaceae_1648	92.75
p__Proteobacteria__g__Rhizobium__s__leguminosarum_2017	91.30
p__Proteobacteria__g__Delftia_3407	79.71
p__Proteobacteria__g__Acinetobacter_4337	76.81
p__Proteobacteria__g__Erwinia_4576	73.91
p__Proteobacteria__g__Pseudomonas_8097	72.46
p__Proteobacteria__g__Staphylococcus_8177	72.46
p__Proteobacteria__f__Enterobacteriaceae_12431	71.01
p__Proteobacteria__g__Sphingomonas_12862	71.01
p__Proteobacteria__g__Delftia_13306	69.57
p__Proteobacteria__g__Bacillus__s__flexus_14993	68.12
p__Proteobacteria__g__Sphingomonas__s__yabuuchiae_17824	68.12
p__Proteobacteria__g__Klebsiella_20803	68.12
p__Proteobacteria__g__Enterococcus_22758	63.77
p__Proteobacteria__g__Propionibacterium__s__acnes_23146	63.77
p__Proteobacteria__g__Ralstonia_23680	57.97
p__Proteobacteria__g__Lysinibacillus__s__boronitolerans_27244	56.52
p__Proteobacteria__g__Agrobacterium_36803	55.07
p__Proteobacteria__f__Oxalobacteraceae_37375	52.17

### Sex Influence on Gut Microbiota Composition

**FIGURE 4:**
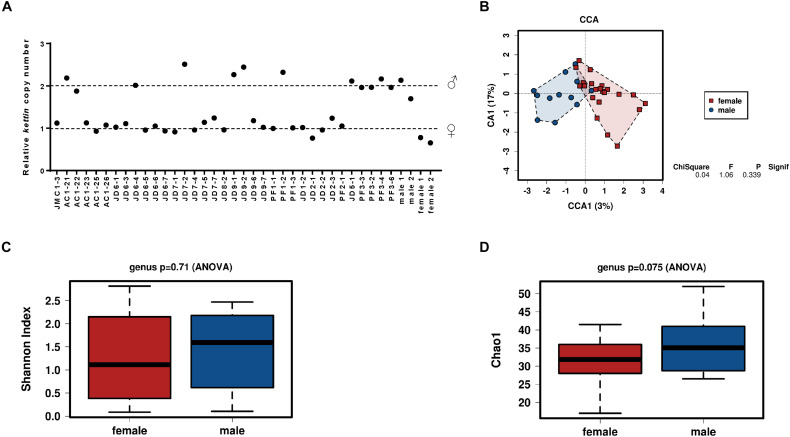
Influence of sex on the microbiota of *S. exigua*. Sex determination of the *S. exigua* larvae from field by quantification of the *kettin* gene copy number **(A)**. Canonical correspondence analysis **(B)**, the estimation of the Shannon index diversity **(C)** and the Chao1 index for richness **(D)** at a genus level according the sex of the collected larvae.

Although caterpillars do not show obvious sex dimorphism, the immature reproductive organs are already present at the larval stage. We wondered if caterpillar sexual destination could influence microbiota composition. To test such hypothesis, the gut microbiota composition from field collected *S. exigua* larvae were also analyzed according to their sex. Sex determination was performed by *kettin* gene quantification in field samples, and 12 males and 23 females were identified (5 samples could not be sexed due lack of proper DNA) (Figure 4A). The bacterial community was examined using a CCA at the genus level to assess if larvae microbiota differs depending on sex, but no significant differences were found (Figure 4B). Additionally, the diversity and richness indices also did not show differences between sexes (Figures 4C,D).

### Gut Bacterial Composition and Viral Infections

**FIGURE 5:**
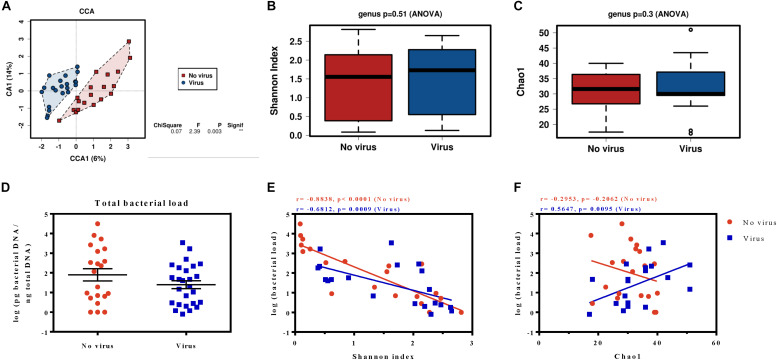
Microbiota analysis of *S. exigua* larvae according their viral infection status. Canonical correspondence analysis **(A)**, the estimation of the Shannon index diversity **(B)** and the Chao1 richness index **(C)** at a genus level in field *S. exigua* larvae naturally infected and non-infected with viruses. Calculation of the total bacterial load of *S. exigua* larvae from viral-infected and viral-free field samples **(D)** and their relationship with diversity **(E)** and richness **(F)**. The best-fit lines, the Spearman r correlations and the p-values of the regression are shown for each analysis.

Naturally present viruses in the *S. exigua* field samples were determined by qPCR amplification of specific viral genes. We found an infectious rate around 50%, which was distributed as follows: 6 larvae infected with BV, 9 larvae infected with SeIV1, and 6 larvae simultaneously infected by both viruses, whereas 19 larvae were classified as virus-free. The gut microbiota was then examined at the genus level according to the presence or absence of viral infection. Although the Shannon and the Chao1 indices estimated that diversity and richness, respectively, were roughly the same for virus-infected and non-virus-infected samples (Figures 5B,C), the CCA analysis showed a significant difference (*P* = 0.003) in microbiota composition (Figure 5A).

An additional analysis revealed the existence of 9 OTUs significantly associated (*P* < 0.05) and more abundant (at least twice) on the viral infected samples (Table 2 and Supplementary Figure 2), the sequences of which are found in the supplementary data. Six of the identified OTUs belong to phylum Proteobacteria (three from the genus *Acinetobacter*), two OTUs were identified as bacteria from genus *Pedobacter* (phylum Bacteroidetes), and another from phylum Firmicutes. An interesting observation is that all OTUs which showed significant abundance changes were overrepresented in the virus-infected group. A moderate increase in abundance of less than 10-fold was found for all the identified OTUs, except for the p__*Proteobacteria*__g__*Acinetobacter*_12048 which is 36,120-fold more abundant in the virus-infected group. Nevertheless, no differences in bacterial load were observed between the viral-infected and non-infected caterpillars (Figure 5D). Interestingly, a deeper analysis revealed a significant negative correlation between the bacterial load and the diversity index for both groups (Figure 5E). However, the relationship between bacterial load and richness only showed a significant positive correlation for the viral infected samples (Figure 5F), which suggests that an increase in microbiota in viral-infected samples leads to the presence of a greater number of bacterial taxa.

**TABLE 2:** Differentially abundant OTUs associated to viral infection.

Otu	Fold change	*P*-valor
p__Bacteroidetes__g__Pedobacter_3455	2.1	0.0020
p__Bacteroidetes__g__Pedobacter_1816	2.8	0.0038
p__Proteobacteria__g__Acinetobacter__s__johnsonii_15272	9.5	0.0065
p__Proteobacteria__g__Acinetobacter_12048	36,120	0.0270
p__Proteobacteria__g__Acinetobacter_15052	3.1	0.0330
p__Firmicutes__g__Staphylococcus_1362	9.9	0.0250
p__Proteobacteria__g__Sphingobium_40602	2.8	0.0250
p__Proteobacteria__f__Xanthomonadaceae_11563	2.8	0.0092
p__Proteobacteria__f__Enterobacteriaceae_14126	3.2	0.0370

## Discussion

In this work, we performed a comprehensive characterization of *S. exigua* larvae gut bacterial microbiota composition, and determined how aspects such as diet, location, sex, and viral presence influence it. Taxonomic analysis revealed that the *S. exigua* gut bacterial community independent of the feeding conditions (diet and origin), was mainly composed of Proteobacteria, Firmicutes, Bacteroidetes, and Actinobacteria, as previously described by Gao et al. (2019). These four phyla were also the most common found in other lepidopteran species (Whitaker et al., 2016; Xia et al., 2017; Chen et al., 2018; Phalnikar et al., 2018; van Schooten et al., 2018), as well as in other insects (Yun et al., 2014). Slight abundance differences were observed between the different groups tested, but likely the main difference was the increased presence of the phylum Firmicutes in larvae reared on AD in comparison with those reared on a PBD (Figure 1). This observation suggests that diet could influence *S. exigua* larvae microbial gut composition. Thus, the bacterial composition was further analyzed.

In a more detailed analysis with respect to gut bacterial composition we focused on laboratory colony larvae fed different diet types (AD, tomato (A and MM varieties), and pepper). A rapid shift in gut bacterial composition was associated with diet changes. Although the relative abundance analyses of the most abundant genera showed great heterogeneity and variability, even among samples from the same diet group, a significant difference was observed in the multivariate analysis (CCA) indicating that larval gut microbiota composition is strongly influenced by diet. No significant differences were observed neither in diversity nor in richness at the genus level, however, the samples reared on AD showed great variability, while samples reared on a PBD were more homogeneous (Figure 2). This observation could be because those insects came from different generations collected at different periods. In addition, although the AD recipe was not changed, the ingredients with it is made of can be slightly differ from one batch to another and affect someway the larvae gut microbiota. Other differences between larvae reared on AD or PBD can be extracted from this study. For example, the genus *Enterococcus* and others from the *Enterobacteriaceae* family were more abundant in samples from AD, as previously shown for *S. exigua* and other lepidopteran species (Broderick et al., 2004; Xiang et al., 2006; Raymond et al., 2009; Hernández-Martínez et al., 2010; Jakubowska et al., 2013). However, in these previous studies *Enterococcus* was reported as a bacterium present in all samples as the most abundant, while in our analysis *Enterococcus* genus abundance was highly variable among samples. Although a fast shift in microbiota composition had been observed after only 48 h of shifting diet, we cannot discard that longer time (e.g., at least one complete generation) could show stronger effects. In addition, previous Lepidoptera studies revealed apparent contradictions with respect to diet influence on gut microbiota. Some studies claimed diet significantly influenced lepidopteran gut microbiota composition (Yun et al., 2014; Berman et al., 2018; Phalnikar et al., 2018; Jones et al., 2019), whereas other authors reported diet had little or no effect on microbiota (Whitaker et al., 2016; Chaturvedi et al., 2017; Minard et al., 2019). This apparent contradiction seems to reveal the lack of caterpillar specific gut microbiota as reported by Hammer et al. (2017). Consequently, any diet change (and the microbiota present in the diet) or in the environment would produce a shift in gut microbiota composition without being diet specific.

In agreement with that, field larvae samples also showed great variability in gut bacterial composition, even though the caterpillars were all collected from pepper plants. Nevertheless, some general microbial composition patterns were observed when samples were grouped according to their location (Figure 3), suggesting a stronger contribution from environmental bacteria in the final larval gut composition than bacteria associated with PBD. This is consistent with a recent study by Hannula and colleagues (Hannula et al., 2019), in which they demonstrated that *Mamestra brassicae* larvae microbiota depends on the soil microbiome. So, the variability observed among the different greenhouses could be attributed to differences in foliar microbiota of each pepper plant and the greenhouse soil microbiota in which the caterpillars were collected. This explanation is also supported by previous studies in which the larval gut bacteria did not totally correspond with dietary associated leaf bacteria (Whitaker et al., 2016; Hammer et al., 2017; Minard et al., 2019). Thus, the authors suggested larval gut microbiota is composed of transient bacteria instead of resident microbiota, due to the intrinsic insect gut physiology, such as their high pH, simple tube structure, and rapid digestion that prevent gut colonization by bacterial uptake with food (Hammer et al., 2017). In agreement with this hypothesis, we observed the absence of resident microbiota. Although we found about 20 OTUs present in at least 50% of the samples, we were unable to find a core microbiome, and no OTU was shared by all the analyzed samples. The lack of a resident microbiota was also described in caterpillar from other species such as *Choristoneura fumiferana* (Landry et al., 2015), and also by Hammer et al. (2017) in a study analyzing the gut microbiota of several Lepidoptera species.

Several Lepidoptera studies revealed differences in microbiota community between male and female adults. *Spodoptera litorallis* adults differ in their microbiota community composition, since female microbiota is composed mainly of *Enterococcus*, *Klebsiella*, and *Pantoea* genera, while the male microbiota is dominated by *Klebsiella* (Chen et al., 2016). van Schooten et al. (2018) also reported significant abundance differences between sexes in several *Heliconius* species, but only for 13 rare OTUs, as similarly occurs for a few *Melitaea cinxia* larvae taxa (Minard et al., 2019). Recent *S. exigua* studies by Gao et al. (2019) described the absence of differences in microbiota composition between male and female adults. In our study we compare for the first time the microbiota composition between males and females at the larval stage, and the results showed no sex influence on caterpillar gut bacterial composition, diversity, or richness (Figure 4). Whether this lack of differences can be attributed to the larval stage or to the studied species would need further investigation.

Previous studies in our laboratory revealed that baculovirus infection increases the gut microbiota load in *S. exigua* larvae, and that increase also benefits the virus, enhancing their virulence, pathogenicity, and dispersion in laboratory conditions (Jakubowska et al., 2013). However, the relationship between viral infections in the field and microbiota composition had never been addressed. As occurs for many lepidopteran field populations (Williams et al., 2017), *S. exigua* field insects are naturally infected by different viruses (Virto et al., 2014). This is the first time that SeMNPV and/or SeIV1 presence was related with *S. exigua* larvae microbiota composition. Interestingly, although no differences were observed in diversity or richness, the results obtained with the multivariant analysis carried out with field samples showed a different gut bacterial composition associated with the presence or absence of viral infections (Figure 5). The bacterial load also did not show differences for infected and non-infected field larvae. However, a negative correlation between bacterial load and diversity levels was observed for both groups. So, the greater total bacterial loads in the *S. exigua* gut can be explained by the increased abundance of only a few bacterial groups, as occurs with *Enterococcus* in laboratory *S. exigua* populations (Jakubowska et al., 2013). In the case of richness, a positive correlation was observed only for the viral-infected samples, which means that the larval microbiota is composed of a greater number of bacterial species. In addition, significant abundance differences were observed for 9 OTUs, which were always more prevalent in infected *S. exigua* larvae than in the virus-free samples. Half of these OTUs belong to genera *Acinetobacter* and *Pedobacter*, suggesting these bacterial genera could have active functions in viral-host interaction. *Staphylococcus*, *Sphingobium*, and unclassified bacteria from families *Xanthomonadaceae* and *Enterobacteriaceae* are also differentially abundant OTUs when larvae are virally infected.

*Wolbachia* is an intracellular bacterium commonly found in insects, and present in about 80% of lepidopteran species (Ahmed et al., 2015). Graham et al. (2012) reported that *Wolbachia* increased *S. exempta* susceptibility to baculovirus, becoming a potential biological control agent. However, it was absent from our field larvae samples. *Wolbachia* is usually found in reproductive tissues, and was described as a parasite that manipulates reproduction in Lepidoptera (Hiroki et al., 2002; Werren et al., 2008), but it can also be found in other tissues, even in the gut (Narita et al., 2007; Whitaker et al., 2016). Since our work focused on gut microbiota, we cannot discard *Wolbachia* presence in other larval tissues. Nevertheless, our study identified certain bacterial groups that could influence the infection cycle, and perhaps increase susceptibility to viral infections, or even trigger covert viral activation, which could be key to developing new pest control strategies through insect microbiota manipulation.

## Conclusion

*S. exigua* larvae microbiota is mainly composed of Proteobacteria, Firmicutes, Bacteroidetes, and Actinobacteria phyla. Although some differences in gut bacterial composition were observed according the different diets analyzed, the results showed that gut microbiota is highly variable and strongly influenced by environmental bacteria (including those acquired through diet), supporting previous studies reflecting the lack of resident microbiota in caterpillars. In addition, microbiota study related to field larvae natural infection status revealed that the microbiota composition is significantly different when larvae present covert infections, and individual taxa could be associated specifically with the infection. Our observations suggested the possibility that microbiota composition influences the infective process of these *S. exigua* larvae pathogens. Thus, these results offer valuable information that will be useful for insect pest management using entomopathogenic viruses.

## Data Availability Statement

The raw Illumina sequences generated and analyzed for this study can be found in the NCBI Sequence Read Archive (SRA) under BioProject number PRJNA603888.

## Author Contributions

MM-S and SH designed the project. MM-S conducted the research. MM-S and MC analyzed the data. MM-S and SH wrote the manuscript.

## Conflict of Interest

The authors declare that the research was conducted in the absence of any commercial or financial relationships that could be construed as a potential conflict of interest.
