# Corrigendum: Influence of Diet, Sex, and Viral Infections on the Gut Microbiota Composition of *Spodoptera exigua* Caterpillars

**DOI:** 10.3389/fmicb.2020.619955

**Published:** 2020-11-16

**Authors:** María Martínez-Solís, María Carmen Collado, Salvador Herrero

**Affiliations:** ^1^Estructura de Recerca Interdisciplinar en Biotecnologia i Biomedicina (ERI BIOTECMED), Departamento de Genética, Universitat de València, Valencia, Spain; ^2^Instituto de Agroquímica y Tecnología de Alimentos, Consejo Superior de Investigaciones Científicas (IATA-CSIC), Valencia, Spain

**Keywords:** lepidoptera, microbiota, *Spodoptera exigua*, 16S rRNA, viral infection

There is an error in the Funding statement. The correct number for **VIROPLANT project which has received funding from the European Union's Horizon 2020 Research and Innovation Program** is **773567**.

The authors apologize for this error and state that this does not change the scientific conclusions of the article in any way. The original article has been updated.

