# Supplementary material for: Influence of Diet, Sex, and Viral Infections on the Gut Microbiota Composition of Spodoptera exigua Caterpillars
Source: Front Microbiol. 2020 May 6;11:753. doi: 10.3389/fmicb.2020.00753 (PMC7218101; doi:10.3389/fmicb.2020.00753)
Supplement: Supplementary file 1 [file Data_Sheet_1.docx]

Supplementary Material

# Supplementary Tables

**Supplementary Table 1.** Primer list.

| Primer name | Sequence (5’-3’) |
| --- | --- |
| 16S amplicon F | TCGTCGGCAGCGTCAGATGTGTATAAGAGACAGCCTACGGGNGGCWGCAG |
| 16S amplicon R | GTCTCGTGGGCTCGGAGATGTGTATAAGAGACAGGACTACHVGGGTATCTAATCC |
| Se kettin_qPCR_F | AGCACGATGTGACGCCGAGTGT |
| Se kettin_qPCR_R | ATTGCTTGACCCTCGGTAACAA |
| Se_BV_qF | ACAGCTTTCCCATCGTCAAC |
| Se_BV_qR | AATCGGAAGCAACGGTTAGG |
| Se_IV1_qF | TGTGAAGTTAGACACGCATGGAA |
| Se_IV1_qR | CGACTTGTGCTACTCTCTTCATCAA |
| ATPsyn-F | TCCTGCTGTTGTTCGCTTTC |
| ATPsyn-R | CCACACATTCGATTCCATGGC |

**Supplementary Table 2.** List of the *S. exigua* analyzed samples and their number of reads.

| **Sample origin** | **Sample code** | **Raw reads** | **Cleaned reads^a^** | **Filtered reads^b^** |
| --- | --- | --- | --- | --- |
| Field | AC1-21 | 60797 | 56354 | 54627 |
|  | AC1-22 | 65203 | 60619 | 57086 |
|  | AC1-23 | 45170 | 41619 | 35990 |
|  | AC1-25 | 17117 | 15516 | 14388 |
|  | AC1-26 | 32715 | 30004 | 27557 |
|  | JD1-1* | 6346 | 2345 | 280 |
|  | JD1-2 | 18341 | 6524 | 1008 |
|  | JD2-1 | 57463 | 28738 | 5373 |
|  | JD2-2 | 66702 | 52124 | 38728 |
|  | JD2-3 | 20799 | 14706 | 1292 |
|  | JD5-1 | 17914 | 12447 | 5824 |
|  | JD5-2 | 19131 | 14446 | 2053 |
|  | JD5-3* | 14243 | 8134 | 789 |
|  | JD6-1 | 68782 | 64381 | 61593 |
|  | JD6-3 | 88388 | 82705 | 81968 |
|  | JD6-4 | 252166 | 238930 | 236334 |
|  | JD6-5 | 78837 | 74369 | 73752 |
|  | JD6-6 | 93257 | 88048 | 87266 |
|  | JD6-7 | 75226 | 71453 | 70440 |
|  | JD7-1 | 62561 | 59188 | 58501 |
|  | JD7-2 | 111393 | 105207 | 104521 |
|  | JD7-4 | 78946 | 72626 | 69756 |
|  | JD7-5 | 41694 | 37860 | 37077 |
|  | JD7-7 | 31596 | 30079 | 29850 |
|  | JD8-2 | 71328 | 59963 | 29923 |
|  | JD9-1 | 91241 | 86736 | 84372 |
|  | JD9-2 | 56794 | 52912 | 36514 |
|  | JD9-6 | 57918 | 54756 | 54236 |
|  | JD9-7 | 76498 | 70953 | 64923 |
|  | JMC1-1 | 28966 | 17245 | 1818 |
|  | JMC1-2* | 17779 | 11563 | 803 |
|  | JMC1-3 | 27262 | 17998 | 1802 |
|  | JMC1-4 | 34933 | 29593 | 1887 |
|  | JMC1-5* | 57395 | 50378 | 941 |
|  | PF1-1 | 33603 | 16038 | 3823 |
|  | PF1-2 | 67121 | 51266 | 21114 |
|  | PF1-3 | 34089 | 20135 | 10379 |
|  | PF1-8* | 69616 | 63042 | 734 |
|  | PF2-1 | 31064 | 27733 | 16821 |
|  | PF3-1 | 26610 | 23367 | 22551 |
|  | PF3-2 | 10198 | 8725 | 2593 |
|  | PF3-3 | 37168 | 31488 | 1927 |
|  | PF3-4 | 21361 | 19059 | 12534 |
|  | PF3-5 | 84469 | 74890 | 8451 |
|  | PF3-6 | 127338 | 113582 | 22005 |
| Artificial diet | DA1-1 | 15814 | 10621 | 5051 |
|  | DA1-2 | 11233 | 6113 | 3528 |
|  | DA1-3 | 14151 | 7209 | 3512 |
|  | DA3-1 | 37973 | 24649 | 19476 |
|  | DA3-2 | 22897 | 12407 | 8062 |
|  | DA3-3 | 30376 | 17030 | 10600 |
|  | CTR4 | 89907 | 80346 | 77459 |
|  | CTR5 | 43279 | 33193 | 27876 |
|  | CTR6 | 66416 | 50375 | 42504 |
|  | IV1 | 79207 | 69826 | 65440 |
|  | IV2 | 67449 | 60949 | 58904 |
|  | IV3 | 45256 | 36138 | 28835 |
|  | S_IV1 | 34682 | 15446 | 1974 |
|  | S_IV2 | 48205 | 35305 | 26433 |
|  | S_IV3* | 15738 | 7236 | 541 |
| Pepper | P1-1 | 14325 | 7514 | 3118 |
|  | P1-2 | 19916 | 9119 | 2832 |
|  | P1-3 | 16410 | 6825 | 2329 |
|  | P3-1 | 18299 | 8168 | 1522 |
|  | P3-2 | 12809 | 5026 | 1908 |
|  | P3-3* | 5791 | 2648 | 960 |
| Tomato (Ailsa) | A1-1 | 20569 | 11906 | 7387 |
|  | A1-2 | 30547 | 16823 | 6610 |
|  | A1-3 | 28773 | 12212 | 5082 |
|  | A3-1 | 35385 | 20188 | 8177 |
|  | A3-3 | 29160 | 13102 | 4066 |
| Tomato (Money Maker) | M1-1 | 20070 | 12045 | 9150 |
|  | M1-2 | 17063 | 10152 | 5867 |
|  | M1-3 | 28092 | 19589 | 9027 |
|  | M3-1 | 21527 | 7752 | 1933 |
|  | M3-2 | 15859 | 8034 | 4276 |
|  | M3-3* | 5368 | 2448 | 673 |

^a^ number of trimmed and cleaned reads that pass the minimum length or the quality control assessment described in the material and methods section.

^b^ number of reads included in the analysed otu table containing only sequences from the four most abundant phyla after filtering the Unassigned, Mitochondria, Chloroflexi and Cyanobacteria taxa.

*sequences removed from the analyses since they are less than 1000 reads.

**Supplementary Table 3.** Statistics of analyzed reads from the 77 *S. exigua* samples sequenced.

| **Raw reads** | **Analyzed reads** | **Reads per sample** | | | |
| --- | --- | --- | --- | --- | --- |
|  |  | **Mean** | **SD** | **Min.** | **Max.** |
| 3,450.084 | 2,005.595 | 29.067 | 37.411 | 1.008 | 23.6334 |

# Supplementary Data

Representative sequences of the differentially abundant OTUs associated to viral infection:

>p__Bacteroidetes__g__Pedobacter_3455

CCTACGGGAGGCAGCAGTAAGGAATATTGGTCAATGGAGGCAACTCTGAACCAGCCATGCCGCGTGCAGGAAGACGGCCCTCTGGGTTGTAAACTGCTTTTATTCGGGAATAAACCTTTGCTCGTGAGCGAAGCTGAATGTACCGAAGGAATAAGGATCGGCTAACTCCGTGCCAGCAGCCGCGGTAATACGGAGGATCCAAGCGTTATCCGGATTTATTGGGTTTAAAGGGTGCGTAGGCGGCCTGTTAAGTCAGGGGTGAAAGACGGTAGCTCAACTATCGCAGTGCCCTTGATACTGATGGGCTTGAATGGACTAGAGGTAGGCGGAATGAGACAAGTAGCGGTGAAATGCATAGATATGTCTCAGAACACCGATTGCGAAGGCAGCTTACTATGGTCTTATTGACGCTGAGGCACGAAAGCGTGGGGATCGAACAGGATTAGATACCCCAGTAGTC

>p__Bacteroidetes__g__Pedobacter_1816

CCTACGGGTGGCTGCAGTAAGGAATATTGGTCAATGGAGGCAACTCTGAACCAGCCATGCCGCGTGCAGGAAGACGGCCCTCTGGGTTGTAAACTGCTTTTATTCGGGAATAAACCTGCTTACGTGTAAGTAGCTGAATGTACCGAAGGAATAAGGATCGGCTAACTCCGTGCCAGCAGCCGCGGTAATACGGAGGATCCAAGCGTTATCCGGATTTATTGGGTTTAAAGGGTGCGTAGGCGGCCTGTTAAGTCAGGGGTGAAAGACGGTAGCTCAACTATCGCAGTGCCCTTGATACTGATGGGCTTGAATGGACTAGAGGTAGGCGGAATGAGACAAGTAGCGGTGAAATGCATAGATATGTCTCAGAACACCGATTGCGAAGGCAGCTTACTATGGTCTTATTGACGCTGAGGCACGAAAGCGTGGGGATCAAACAGGATTAGATACCCTTGTAGTC

>p__Proteobacteria__g__Acinetobacter__s__johnsonii_15272

CCTACGGGGGGCTGCAGTGGGGAATATTGGACAATGGGCGCAAGCCTGATCCAGCCATGCCGCGTGTGTGAAGAAGGCCTTTTGGTTGTAAAGCACTTTAAGCGAGGAGGAGGCTACTGAGATTAATACTCTTGGATAGTGGACGTTACTCGCAGAATAAGCACCGGCTAACTCTGTGCCAGCAGCCGCGGTAATACAGAGGGTGCGAGCGTTAATCGGATTTACTGGGCGTAAAGCGTGCGTAGGCGGCTTTTTAAGTCGGATGTGAAATCCCTGGGCTTAACTGAGGAATTGCATTCGATACTGGGAAGCTAGAGTATGGAAGAGGATGATAGAATTCCAGGTGTAGCGGTGAAATGCGTAGAGATCTGGAGGAATACCGATGGCGAAGGCAGCCATCTGGCCTAATACTGACGCTGAGGTACGAAAGCATGGGGAGCAAACAGGATTAGATACCCTGGTAGTC

>p__Proteobacteria__f__Xanthomonadaceae_11563

CCTACGGGTGGCAGCAGTGGGGAATATTGGACAATGGGCGCAAGCCTGATCCAGCCATACCGCGTGGGTGAAGAAGGCCTTCGGGTTGTAAAGCCCTTTTGTTGGGAAAGAAAAGCAGTCGGTTAATACCCGGTTGTTCTGACGGTACCCAAAGAATAAGCACCGGCTAACTTCGTGCCAGCAGCCGCGGTAATACGAAGGGTGCAAGCGTTACTCGGAATTACTGGGCGTAAAGCGTGCGTAGGTGGTTGTTTAAGTCTGTTGTGAAAGCCCTGGGCTCAACCTGGGAATTGCAGTGGATACTGGGCGACTAGAGTGTGGTAGAGGGTAGTGGAATTCCTGGTGTAGCAGTGAAATGCGTAGAGATCAGGAGGAACATCCATGGCGAAGGCAGCTACCTGGACCAACACTGACACTGAGGCACGAAAGCGTGGGGAGCAAACAGGATTAGATACCCCTGTAGTC

>p__Firmicutes__g__Staphylococcus_1362

CCTACGGGTGGCAGCAGTAGGGAATCTTCCGCAATGGGCGAAAGCCTGACGGAGCAACGCCGCGTGAGTGATGAAGGTTTTCGGATCGTAAAACTCTGTTATTAGGGAAGAACAAATGCGTAAGTAACTGTGCGCATCTTGACGTTACCTAATCAGAAAGCCACGGCTAACTCCGTGCCAGCAGCCGCGGTAATACGTAGGTGGCAAGCGTTATCCGGAATTATTGGGCGTAAAGCGCGCGTAGGCGGTTTCTTAAGTCTGATGTGAAAGCCCACGGCTCAACCGTGGAGGGTCATTGGAAACTGGGAAACTTGAGTGTAGTAGAGGAAAGTGGAATTCCATGTGTAGCGGTGAAATGCGCAGAGATATGGAGGAACACCAGTGGCGAAGGCGACCTTCTGGTCTGTAACTGACGCTGATGTGCGAAAGCGTGGGGATCAAACAGGATTAGATACCCTTGTAGTC

>p__Proteobacteria__g__Sphingobium_40602

CCTACGGGAGGCAGCAGTAGGGAATATTGGACAATGGGCGAAAGCCTGATCCAGCAATGCCGCGTGAGTGATGAAGGCCTTAGGGTTGTAAAGCTCTTTTACCCGGGATGATAATGACAGTACCGGGAGAATAAGCTCCGGCTAACTCCGTGCCAGCAGCCGCGGTAATACGGAGGGAGCTAGCGTTGTTCGGAATTACTGGGCGTAAAGCGCACGTAGGCGGCTATTCAAGTCAGAGGTGAAAGCCCGGGGCTCAACCCCGGAACTGCCTTTGAAACTAGATAGCTTGAATCCAGGAGAGGTGAGTGGAATTCCGAGTGTAGAGGTGAAATTCGTAGATATTCGGAAGAACACCAGTGGCGAAGGCGGCTCACTGGACTGGTATTGACGCTGAGGTGCGAAAGCGTGGGGAGCAAACAGGATTAGATACCCCAGTAGTC

>p__Proteobacteria__g__Acinetobacter_12048

CCTACGGGAGGCAGCAGTGGGGAATATTGGACAATGGGGGGAACCCTGATCCAGCCATGCCGCGTGTGTGAAGAAGGCCTTTTGGTTGTAAAGCACTTTAAGCGAGGAGGAGGCTACCGAGATTAATACTCTTGGATAGTGGACGTTACTCGCAGAATAAGCACCGGCTAACTCTGTGCCAGCAGCCGCGGTAATACAGAGGGTGCAAGCGTTAATCGGATTTACTGGGCGTAAAGCGCGCGTAGGTGGCCAATTAAGTCAAATGTGAAATCCCCGAGCTTAACTTGGGAATTGCATTCGATACTGGTTGGCTAGAGTATGGGAGAGGATGGTAGAATTCCAGGTGTAGCGGTGAAATGCGTAGAGATCTGGAGGAATACCGATGGCGAAGGCAGCCATCTGGCCTAATACTGACACTGAGGTGCGAAAGCATGGGGAGCAAACAGGATTAGATACCCTGGTAGTC

>p__Proteobacteria__g__Acinetobacter_15052

CCTACGGGAGGCAGCAGTGGGGAATATTGGACAATGGGGGGAACCCTGATCCAGCCATGCCGCGTGTGTGAAGAAGGCCTTATGGTTGTAAAGCACTTTAAGCGAGGAGGAGGCTCTTCTAGTTAATACCTAGGATGAGTGGACGTTACTCGCAGAATAAGCACCGGCTAACTCTGTGCCAGCAGCCGCGGTAATACAGAGGGTGCGAGCGTTAATCGGATTTACTGGGCGTAAAGCGTGCGTAGGCGGCTTTTTAAGTCGGATGTGAAATCCCCGAGCTTAACTTGGGAATTGCATTCGATACTGGGAAGCTAGAGTATGGGAGAGGATGGTAGAATTCCAGGTGTAGCGGTGAAATGCGTAGAGATCTGGAGGAATACCGATGGCGAAGGCAGCCATCTGGCCTAATACTGACGCTGAGGTACGAAAGCATGGGGAGCAAACAGGATTAGATACCCCAGTAGTC

> p__Proteobacteria__f__Enterobacteriaceae_14126

CCTACGGGTGGCAGCAGTGGGGAATATTGCACAATGGGCGCAAGCCTGATGCAGCCATGCCGCGTGTATGAAGAAGGCCTTCGGGTTGTAAAGTACTTTCAGTCAGGAGGAAGGGTGTGAAATTAATACTTTCATGCATTGACGTTACTGACAGAAGAAGCACCGGCTAACTCCGTGCCAGCAGCCGCGGTAATACGGAGGGTGCAAGCGTTAATCGGAATTACTGGGCGTAAAGCGCACGCAGGCGGTTTGTTAAGTCAGATGTGAAATCCCCGGGCTCAACCTGGGAACTGCATTTGAAACTGGCAAGCTTGAGTCTTGTAGAGGGGGGTAGAATTCCAGGTGTAGCGGTGAAATGCGTAGAGATCTGGAGGAATACCGGTGGCGAAGGCGGCCCCCTGGACAAAGACTGACGCTCAGGTGCGAAAGCGTGGGGAGCAAACAGGATTAGATACCCTGGTAGTC

# Supplementary Figures

#
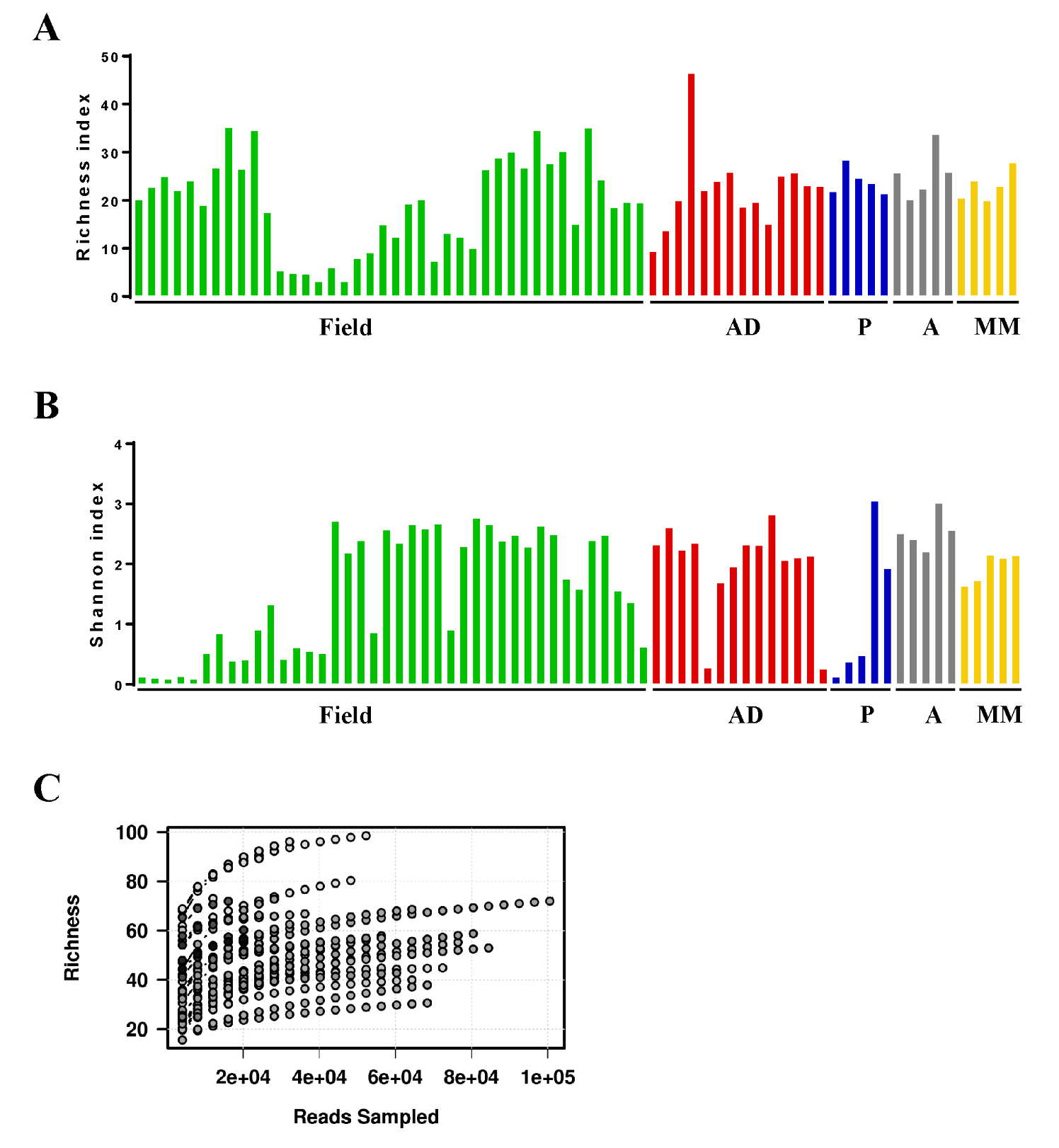


**Supplementary Figure 1.** Richness and diversity of analyzed *S. exigua* samples. Bar charts representing the Richness index **(A)** and the diversity Shannon index **(B)** of individual samples. OTU rarefaction curves of individual samples **(C)**. A field sample with more than 200,000 reads (JD6-4) was removed from the rarefaction curves plot because it was out of scale.

**
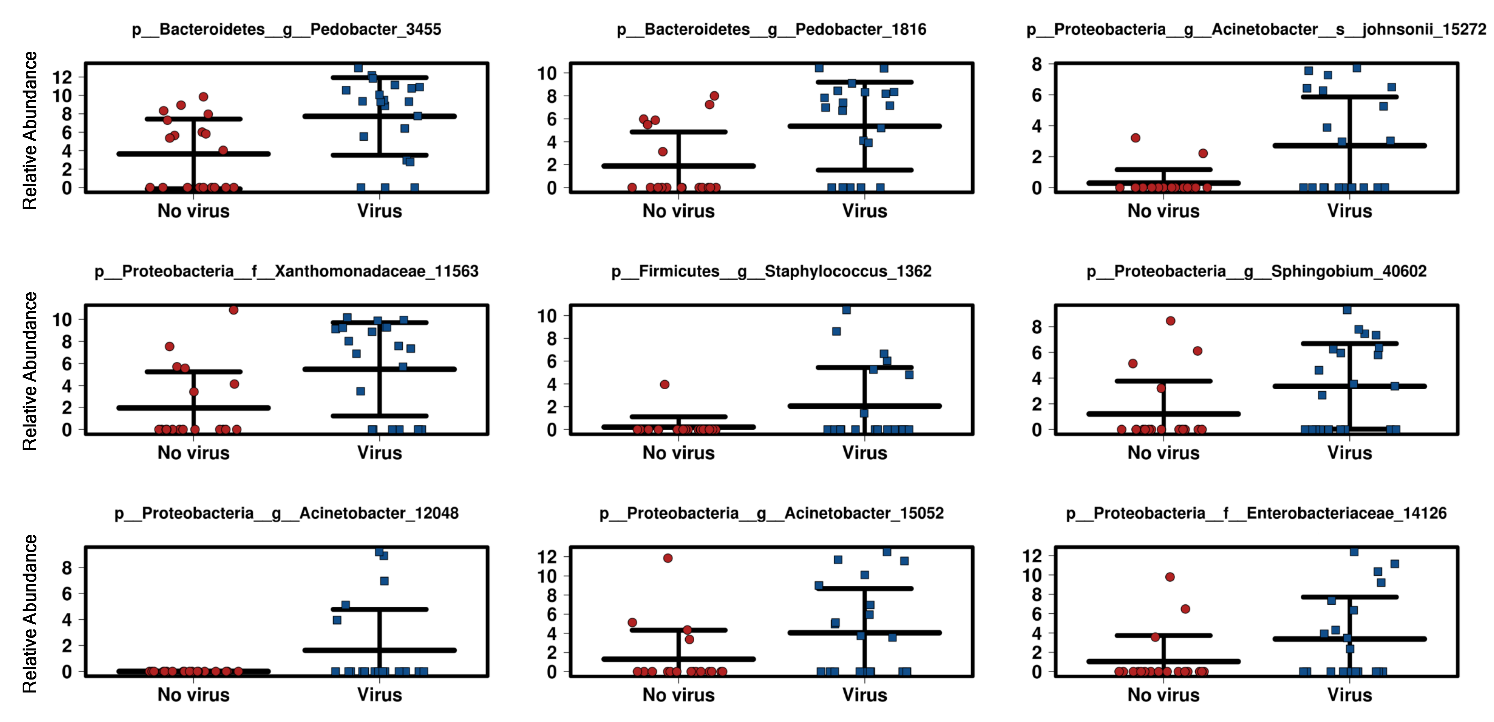
**

**Supplementary Figure 2.** Representation of the relative abundances of the statistically significant different OTUs associated to viral infections.
